# Use of Biometric Images to Predict Body Weight and Hot Carcass Weight of Nellore Cattle

**DOI:** 10.3390/ani13101679

**Published:** 2023-05-18

**Authors:** Alexandre Cominotte, Arthur Fernandes, João Dórea, Guilherme Rosa, Rodrigo Torres, Guilherme Pereira, Welder Baldassini, Otávio Machado Neto

**Affiliations:** 1 Department of Animal Science, University of Wisconsin, Madison, WI 53706, USA; 2 School of Agricultural and Veterinarian Sciences, São Paulo State University, Jaboticabal 14884-900, SP, Brazil; 3 Department of Biostatistics and Medical Informatics, University of Wisconsin, Madison, WI 53706, USA; 4 School of Veterinary and Animal Science, São Paulo State University, Botucatu 18618-681, SP, Brazil

**Keywords:** image analysis, beef cattle, Kinect^®^, models, computer vision

## Abstract

**Simple Summary:**

Body and carcass weight are important economic characteristics for beef cattle production systems because the value of market cattle is based on weight. The possibility of predicting body and carcass weight through biometric measurements obtained from three-dimensional digital images favor the development of the production system. Predictive approaches, such as artificial neutral network, showed better predictive quality for body weight, while the least absolute shrinkage and selection operator and partial least square models were the most suitable for predicting carcass weight.

**Abstract:**

The objective of this study was to evaluate different methods of predicting body weight (BW) and hot carcass weight (HCW) from biometric measurements obtained through three-dimensional images of Nellore cattle. We collected BW and HCW of 1350 male Nellore cattle (bulls and steers) from four different experiments. Three-dimensional images of each animal were obtained using the Kinect^®^ model 1473 sensor (Microsoft Corporation, Redmond, WA, USA). Models were compared based on root mean square error estimation and concordance correlation coefficient. The predictive quality of the approaches used multiple linear regression (MLR); least absolute shrinkage and selection operator (LASSO); partial least square (PLS), and artificial neutral network (ANN) and was affected not only by the conditions (set) but also by the objective (BW vs. HCW). The most stable for BW was the ANN (Set 1: RMSEP = 19.68; CCC = 0.73; Set 2: RMSEP = 27.22; CCC = 0.66; Set 3: RMSEP = 27.23; CCC = 0.70; Set 4: RMSEP = 33.74; CCC = 0.74), which showed predictive quality regardless of the set analyzed. However, when evaluating predictive quality for HCW, the models obtained by LASSO and PLS showed greater quality over the different sets. Overall, the use of three-dimensional images was able to predict BW and HCW in Nellore cattle.

## 1. Introduction

The body weight is an important economic trait for beef cattle production systems, mainly because it allows the evaluation of individual animal growth, and the value of market cattle is based on weight. Therefore, it is possible to optimize the nutritional management in the different phases of the production cycle [1,2]. However, weighing carried out in the traditional way causes considerable financial losses to producers due to weight loss, a decrease in immunity, and even accidents during this process [3]. This is due to the time animals remain in the handling pen, in most cases, without feed and/or water and reared with other groups. For these reasons, the weighing of beef cattle cannot be carried out routinely or in short intervals of time, which would be very useful within breeding systems for adapting diets during growth and, mainly, in the finishing phase. 

A promising approach is to obtain biometric measurements through three-dimensional digital images, which can be correlated with weight or frame size. In recent years, this type of approach has shown promise due to its low cost and possibility of automation, which eliminates financial losses linked to conventional weighing and allows access to weight estimates in real time [4,5]. However, the studies that evaluated image processing in animal production used different approaches and models for prediction, such as linear regression to predict body weight in dairy cattle [4] and beef cattle [6], and the Gaussian mixture model for predicting the body score condition in dairy cows [7].

However, the prediction quality (accuracy and precision) of the models between biometric variables via three-dimensional images and BW and HCW are affected not only by the chosen predictor variables [8] but also by multicollinearity characteristics and linear and non-linear relationships with the explanatory variable when using an image to predict body weight [9]. In this context, our hypothesis is that the use of approaches, such as partial least squares regression (PLS) and artificial neural network (ANN), present better predictive quality from variables extracted from the image for the prediction of BW and HCW in beef cattle. The predictive quality of the PLS and ANN methods appears to be less affected by the multicollinearity process between the independent variables [10] and has an adequate prediction ability even for complex relationships between variables in a non-linearity condition [11]. 

In this context, the objective of this study was to use and evaluate different methods of predicting BW and HCW from biometric measurements obtained through three-dimensional images of Nellore cattle.

## 2. Materials and Methods

All the animal procedures were approved by the Committee of Ethics in the use of animals of the School of Agricultural and Veterinarian Sciences of the São Paulo State University, Brazil, protocol number 007946/18.

### 2.1. Dataset and Obtaining Biometric Measurements by Images

#### 2.1.1. Samples Used

For this work, BW data at the end of feedlot period and HCW were collected from 450 male Nellore cattle raised in single batches allocated within four different experiments (Exp; Table 1). Exp. 1 was composed of 48 chemically castrated male Nellore; Exp. 2 consisted of 83 Nellore bulls and Exp. 3 and 4 were composed of 228 and 91 Nellore bulls, respectively. These experiments were conducted in different years at the São Paulo State University (FMVZ UNESP), Botucatu, São Paulo, Brazil.

#### 2.1.2. Collection of Weights and Biometric Measurements by Images

The body and hot carcass weights of the 450 animals were obtained from an electronic scale (Coimma LTDA, model RUDD 300, Dracena, São Paulo, Brazil, with an accuracy of ±0.5 kg) at the end of the feedlot period of each experiment and immediately after slaughter and evisceration. The three-dimensional images of each of these animals and their body weights were obtained at the end of the feedlot period. For this, a Kinect^®^ model 1473 sensor (Microsoft Corporation, Redmond, WA, USA) was used. It was positioned perpendicularly (90° angle) to the ground in the upper part of the cattle chute, respecting the same distance from the ground in all cases. Infrared image capture time (640 × 480 pixels) was 15 s, resulting in 10 frames per animal. Then, the 10 frames obtained for each animal were processed in three steps: (1) estimation of the distance from the ground to the camera, (2) image segmentation, and (3) feature extraction. 

**Figure 1:**
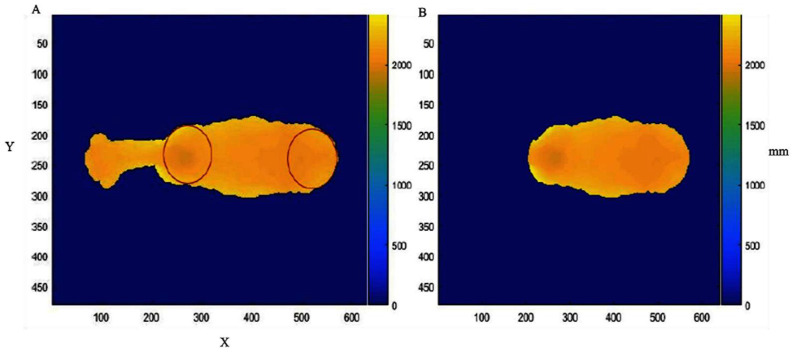
(**A**) Shoulder and rump identified through a Hough transformation adaptation for the identification of round objects; (**B**) image of the animal’s back segmented after the identification of round objects. X and Y correspond to the coordinates of each pixel in the Cartesian plane, and mm represents the distance in millimeters from the camera to each pixel point in the image.

The steps cited above are described by Cominotte et al. [9] and were followed in this work. The best segmented frame was manually selected (one frame per animal by observer). As it is a controlled experiment for model calibration, some steps that are normally automated were performed manually. Therefore, the processing (manual evaluation) was carried out in this step, which consisted of evaluating the inclusion of the animal in the scene and its connectivity with the objects and the removal of these objects. In addition, the position of the animal’s body (and the positioning of the vertebral column) was verified through the outline of the dorsal area that was between the shoulder and the rump. By contrast, the determination of the shoulder and rump positions was carried out through an adaptation of the Hough transformation to identify round objects (Figure 1; [12,13]). After this supervised process, the image segments corresponding to biometric measurements were demarcated and extracted using an algorithm. The set of extracted features could be divided into two classes: body measurements and shape descriptors.

**Figure 2:**
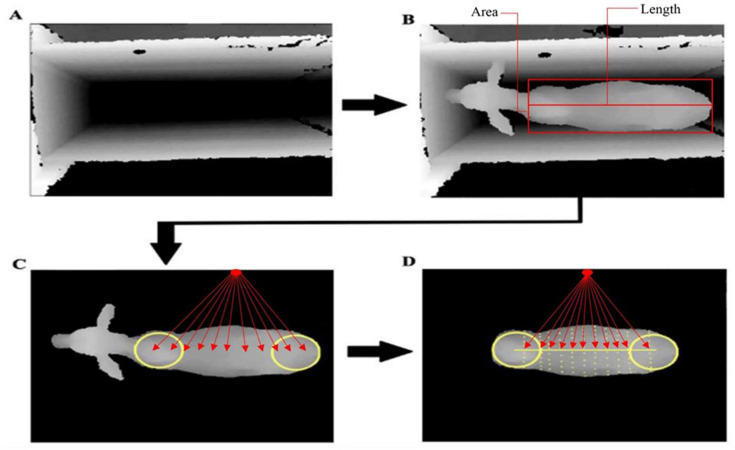
Extraction of body measurements at equidistant locations on the back of the animal, from the shoulders to the rump: (**A**) Containment trunk without the animal; (**B**) area and length measurements, (**C**) height measured at 11 points along the back, and (**D**) body volume (Height × Length × Width) = Width measurements 11 points—dorsal area [yellow line] × Height measured at 11 points along the back [red line].

The body measurements were area, volume, maximum length, width, and height at equidistant locations on the dorsal part of the animal from the shoulders to the rump (Figure 2), and the length, width, and area of an object in an image that could be estimated by the sum of pixels. Fourier descriptors were used in this process in the same way as described by Cominotte et al. [9]. These are global image descriptors commonly used for shape analysis and image matching [14,15,16].

For the weight prediction analysis, the following features were obtained: area (BA) and body volume (BV), eleven widths (W1 to W11) and heights (H1 to H11) along the animal’s back, length, eccentricity, two spinal curvature measurements, and four descriptive Fourier measurements.

### 2.2. Statistical Methods for the Prediciton of Body Weight and Hot Carcass

#### 2.2.1. Correlation and Exclusion of Variables

**Figure 3:**
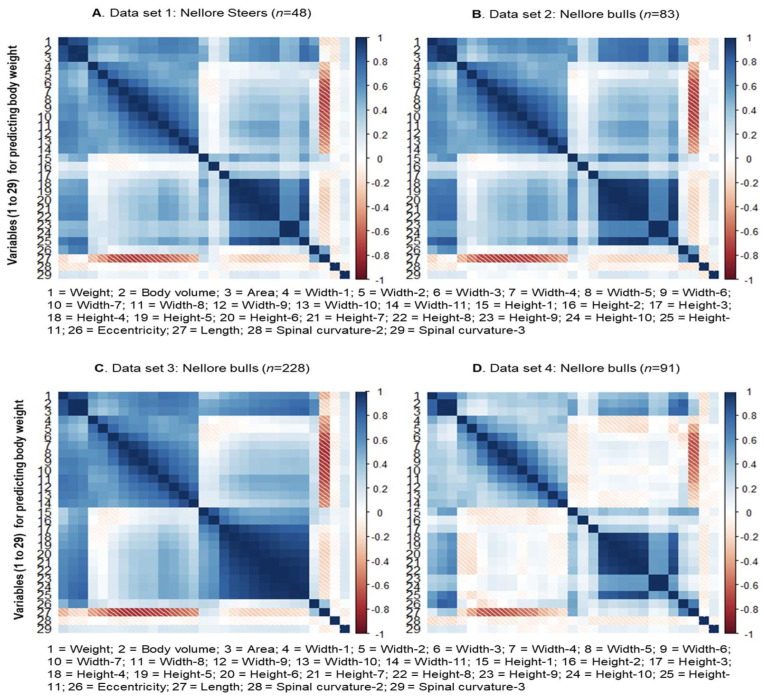
Correlation between the response variable and the explanatory variables for body weight obtained through the Kinect^®^ model 1473 sensor (Microsoft Corporation, Redmond, WA, USA).

**Figure 4:**
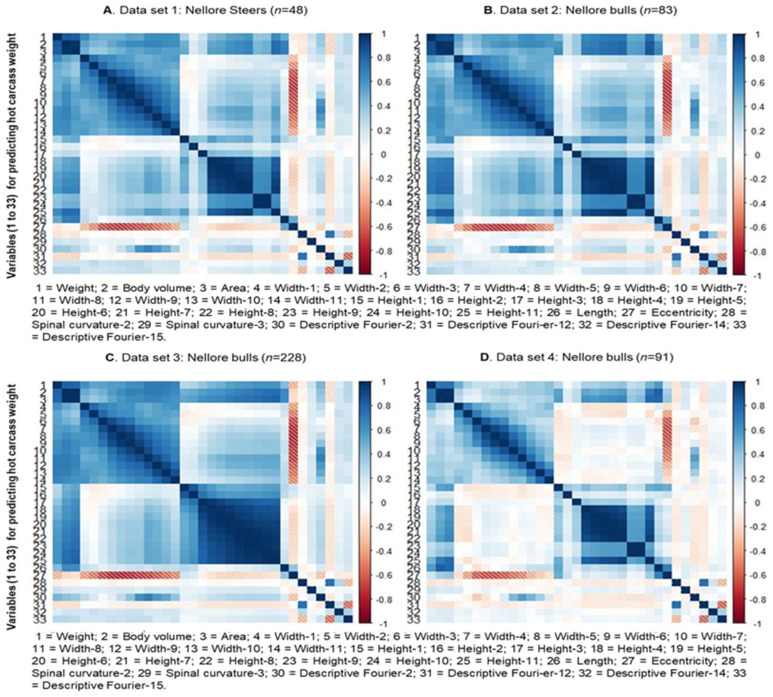
Correlation between the response variable and the explanatory variables for hot carcass weight obtained through the Kinect^®^ model 1473 sensor (Microsoft Corporation, Redmond, WA, USA).

The Pearson correlation coefficients between the animal’s body weight, hot carcass weight, and the characteristics extracted from the three-dimensional images were obtained using the PROC CORR of the SAS 9.4 software (SAS Institute Inc., Cary, NC, USA). Biometric measurements were excluded based on the significance of the correlations (*p* < 0.05). The value of the correlations between the response variable and the 28 explanatory variables for predicting body weight and carcass weight are shown in Figure 3 and Figure 4, respectively.

#### 2.2.2. Formation of Training and Test Sets

For training the different body weight and hot carcass prediction models using three-dimensional measurements, four different batch arrangements (or combinations) were created and used in this work. Thus, different training and tests were created in each of these arrangements. In the first (Set 1), the training set was formed by the union of data from batches 1, 2, and 3. In the second arrangement (Set 2), the training set was formed by batches 1, 2, and 4. In the third arrangement (Set 3), the training set was formed by batches 1, 3, and 4. In the fourth arrangement (Set 4), the training set was formed by batches 2, 3, and 4. To test the predictive models, the sets excluded during the training of each arrangement (set) were used. Therefore, batches 4, 3, 2, and 1 were used to test the models adjusted for data from Set 1, Set 2, Set 3, and Set 4, respectively.

#### 2.2.3. Models Used for Prediction

Prediction of body weight and hot carcass weight was performed at the Department of Animal Sciences at the University of Wisconsin-Madison, United States. Prediction of body weight and hot carcass weight was developed using four different types of prediction methods: (1) artificial neural network, (2) partial least squares, (3) LASSO regression, and (4) multiple linear regression. 

The MLR was performed together with the leave-one-out cross-validation in the software R using the TrainControl function “LOOCV” method from the Caret package (https://cran.r-project.org/web/packages/caret/caret.pdf (accessed on 1 July 2022)), where Y consisted of the animal’s body weightm and the independent variables X were the characteristics obtained through the Kinect^®^ model 1473 sensor (Microsoft Corporation, Redmond, WA, USA).

Partial least squares (PLS) regression was performed with the JMP Pro 13 (SAS Institute Inc., Cary, NC, USA) to predict body weight using the NIPALS predictive variable algorithm. The PLS extracts successive linear combinations of predictors, called factors, which address both the explanation of response variation and the explanation of predictor variation. The number of factors retained in the model was determined using a 10 k-fold validation, with the predicted root mean of the PRESS calculated. The model that reached the lowest PRESS value in the cross-validation was chosen.

The LASSO regression was performed together with the 10 k-fold validation using the cv.glmnet function (nfolds = 10) from the “glmnet” package [17], implemented in the R software. This package implements an algorithm that optimizes model fit parameters using a coordinate descent algorithm. The description of LASSO models developed for body weight prediction and hot carcass weight prediction are available in Supplementary Materials (Tables S1 and S2).

Finally, artificial neural networks (ANN—“artificial neural network”) were applied and adapted from open-source software H_2_O (https://cran.rproject.org/web/packages/h2o/h2o.pdf (accessed on 1 July 2022)) through statistical software R (R Core Team, 2016, Vienna, Austria). The hyperparameters (variables that determine the network structure) used in machine learning can affect how the algorithm fits the data. We used a combination of hyperparameters to predict body weight and hot carcass weight as described by Dórea et al. [18]. After searching the neural network, the hyperparameters for body weight and hot carcass weight (Supplementary Materials Tables S1 and S2) were determined. Each model with its respective artificial neural network structures was trained through 10 k-fold validation. The H20.deeplearning function was used to train the models. ADADELTA was the adaptive learning rate algorithm used in all ANN [19].

### 2.3. Evaluation of Weight Predictions

The model evaluation system was conducted as described by Tedeschi [20]. The mean bias (MB) is probably the most used and the oldest statistic to assess model accuracy. It is calculated by the mean deviation between predicted and observed values [21]. The mean bias represents the error in the central tendency (to assess whether the predicted values are above or below predicted), the slope bias represents errors due to regression, and the random component is the variance that cannot be explained by the linear regression [20]. The mean bias represents the error in central tendency (to assess whether the predicted values are above or below the observed), the slope bias represents errors due to regression, and the random component is the unexplained variance that cannot be explained by linear regression [20].

The coefficient of determination (*r*^2^) was used between the predicted and observed values to define the precision of the equations. The concordance correlation coefficient (CCC) [22], mean bias, and root mean square error prediction (RMSEP) were calculated as a measure of accuracy. The CCC was estimated by Equation (1) [22].

(1) $${\hat{\rho }}_{c}=\hat{\rho }\times C_{b}\;$$

(2) $$r=\frac{cov\left(X,\;Y\right)}{\sigma_{x}\sigma_{y}}$$

where the first component ($\hat{\rho }$) is the correlation coefficient estimate (r; Equation (2) may be computed using the covariance of *X* and *Y* and standard deviation of *x* and *y*) that measures the precision. The second component (*C_b_*) is the polarization correction factor that represents how much the regression line deviates from the slope of the unit (45°). The *C_b_* factor varies from 0 to 1.

The root mean square error prediction (RMSEP) is the mean square error prediction (MSEP). Where MSEP is the sum of the squared difference between the observed and predicted model, and the values are divided by the number of data points [23]. The MSEP was decomposed into mean bias, slope bias, and random errors [24] to provide an indication of the model’s adequacy for prediction. The models were evaluated using the software Model Evaluation System (MES, College Station, TX, USA; http://nutritionmodels.tamu.edu/mes.html, accessed on 1 July 2022) as described by Tedeschi [20].

## 3. Results and Discussion

The biometric measurements obtained through images were selected based on the significance of the correlation with the BW and HCW response variables within each training set (Set 1 to 4). In addition, to mitigate multicollinearity problems, explanatory variables (features) with significant correlation with each other were excluded, keeping, in this case, only the variables with the highest correlation with the response variable.

In this sense, it is worth noting that for both BW and HCW, in some groups of explanatory variables, high positive correlation between them were observed, mainly, between measurements of width (L1 to L11) and between measurements of height (A1 to A11; Figure 3 and Figure 4). This follows what is observed in relation to studies of biometric measurements taken in locu (or conventionally) in beef cattle and other livestock species [6,9].

Furthermore, a strong positive correlation was observed between volume (2) and body area (3) not only with each other but also with the other measurements of height and width, BW and HCW. Above all, it can be highlighted that volume (2) and body area (3) were the measures most strongly correlated with BW and HCW among all those obtained in this study. So far, what was described above was more clearly observed in Sets 1, 2, and 3 and not so evident for Set 4, considering both the prediction scenarios for BW and HCW. The variables selected from the correlation analysis were concatenated to be used as variables for training and testing in all sets (Sets 1, 2, 3, and 4).

Regarding the variables considered for prediction (and training) of BW, it was observed that length (27) tends to have a negative correlation with width measurements in all sets and has no significant correlation with BW. By contrast, the curvatures of the spine (28 and 29) did not present a significant correlation with any other body measurement, volume, or area in all sets; the same occurred with height 2 (16) in Sets 1, 2, and 4.

Regarding the explanatory variables obtained for HCW prediction, the same trends mentioned above were observed for dorsal spinal curvature measurements (27 and 28) and height 2 (16) (Figure 4). Differently from what was described for BW, the length variable (25) showed a high positive correlation with measurements of height, volume, body area, and HCW. Meanwhile, no significant correlation was observed between eccentricity (26) and descriptive Fourier (29 to 32) measures (not obtained for BW prediction) with each other and with any other biometric measure. 

Regarding the predicted values for BW and HCW obtained by the different models tested, differences were observed in terms of precision and, mainly, accuracy in the different sets (Table 2 and Table 3). In general, for BW, better predictions were observed in Set 1 (Means bias < 6.62 kg; RMSEP < 20.95 kg) and worse in Set 4 (Mean bias > 11.88; RMSEP > 33.75). In Set 2, low accuracy of the predicted values for BW was observed in all models tested (Mean bias > −12.19), as it happened with Set 4; however, it was underestimated in Set 2 and overestimated in Set 4 (Table 2). In Set 3, there is an inconsistency between the models regarding the accuracy of the predicted BW (Mean bias between −0.65 to 6.04 kg) in relation to what was observed in the other sets, as well as the precision (RMSEP = 27.23 to 36.87 kg; *r*^2^ = 0.39 to 0.53). These were still better than those found in Set 4. When observing CCC (reproducibility index) that reflects both accuracy and precision at the same time, it was not possible to distinguish great differences between the sets, except for Set 1, being superior (0.72 to 0.74) and similar between the models. It is noteworthy that, in Set 3, the BW predictions obtained by PLS were accurate (Mean Bias = −0.65). However, high dispersion of predicted values is observed, indicating low precision (RMSEP 36.87 kg; *r*^2^ = 0.3; CCC = 0.54).

Putting the methods used for BW prediction into perspective, models that use penalties (shortening), PLS, and LASSO had an average performance in terms of accuracy and precision of BW predictions and, in some cases, surpassed by LMR, which, in turn, was quite unstable in the different scenarios; however, it presented a suitable performance in the general context. However, none of the aforementioned modeling methods was more stable than ANN, which showed adequate accuracy and precision in its predictions, regardless of the analyzed set. In this method, higher percentages of errors due to regression (RE) given in slope bias were observed, indicating that explanatory measures have a smaller effect on the variation of predicted values when compared to that observed with the original response variables, which leads to an understanding that there is a possibility of improvement in the adjustment of this model. 

The structure of the data used for training and testing the models was in Set 1, Exp. 4 as an independent validation set, which had the lowest CV (5.12%) among the Exps. In Sep 4, the Exp. 1 was used to test the predictive model, in which a higher CV (10.55%) was observed. However, the opposite was observed regarding the CV of the datasets used for training the models, in which CVs of 11.40 and 7.94% were found for Sets 1 and 4, respectively. Then, some contrast was found between the training CV and the test CV in the sets with the best and worst BW estimates. However, apparently, the lower CV in the test favored better predictions for BW. It is also noteworthy that the Exp. 1 had a distinct BW range, with minimum and maximum values lower than the other Exps.

Regarding HCW, the best estimates were observed in Set 1 (Means bias < 6.89 kg; RMSEP < 14.06 kg) (Table 3). In Set 1, MLR had the lowest mean bias (0.01 kg) among HCW predictions. As for the BW, the best estimates were observed in Set 1 (Means bias < 6.89 kg; RMSEP < 14.06 kg) (Table 3). In Set 1, MLR had the lowest mean bias (0.01 kg) among HCW predictions. However, a high proportion of errors due to regression (87% of MSEP) and due to disturbances (13% of MSEP) are observed, indicating less adjustment of the training model on the variation of the predicted values. This inaccuracy of the predicted values (*r*^2^ = 0.05; CCC = 0.14) can be misinterpreted if only measures of central tendency (Mean bias) are observed, which may make this model unstable for predictions in new datasets. After such consideration, we cannot observe systematic variations in the other sets (2 to 4), with great alternation between the HCW prediction quality between models. Therefore, it is not possible to highlight a single method that has excelled in all scenarios.

However, estimates obtained by LASSO and PLS showed greater stability in terms of the quality of predictions over the scenarios being more balanced in terms of accuracy and precision of predicted values (Table 3). The accuracy of predictions obtained by ANN varied depending on the sets (*r*^2^ = 0.17 to 0.52; CCC = 0.19 to 0.65); in addition, high average bias values were observed (up to−21.27 kg), indicating low accuracy and a high proportion of errors due to regression (21 to 31% MSEP). The predictions obtained by MLR varied a lot in relation to each set, obtaining the best accuracy of the predicted values in some cases (Set 1 and Set 3), however, quite inaccurate in most cases (*r*^2^ = 0.05 to 0.52; CCC = 0.14 to 0.69). Such results indicate that the penalties used by the LASSO and PLS methods were effective in modeling over all sets, managing to deliver reasonable predictions in all sets, and adjusting well to this type of data.

Our results partially validate the hypothesis that the use of approaches, such as ANN or PLS, would present a higher predictive quality of BW and HCW prediction from three-dimensional images. The ANN showed better predictive quality (precision and accuracy) for the BW in the different sets, which demonstrate better reproducibility of this approach for the prediction of BW. However, ANN showed instability in accuracy and precision in different sets for HCW prediction. However, the PLS approach showed better predictive quality for HCW in the different evaluation conditions (sets). 

Results, such as those found here, are important, as they assess the predictive capacity of different models in a dataset regardless of those used in training. Shetty et al. [25] compared the validation of a model using external datasets. First, a dataset was created by randomly excluding cows from the training set; then, a dataset was created by randomly excluding records. These authors concluded that model predictions were overestimated when records from the same cow were maintained in both datasets (training and validation). According to Dórea et al. [18] the validation of models excluding the entire set is closer to reality, as the models must be used to predict new datasets from farms or herds with different diets, climates, and rearing practices.

To investigate how the structuring of data used for training and test from different farms/experiments (independent validation) could affect the predictive capacity of each model in this experiment, predictions using cross-validation (10-kfold) were also performed on each training set (Table 4). The best scenario (Set 1), for which independent validation obtained better predictions than the cross-validation (10-kfold), was applied to the training set for this same scenario. However, the other scenarios (Set 2, 3, and 4) had less precise/accurate predictions in the independent validations compared to the cross validations for these same scenarios. 

The results obtained show that predicting body weight and hot carcass weight from three-dimensional images becomes an important tool to be used. Such technologies may offer a practical and affordable way to reduce the stress of animals in farms and abattoirs. In this sense, taking into account the reduced size of the sample set, as well as the high variation in accuracy/precision of the weight predictions for BW and HCW in the different proposed scenarios, it was possible to notice that image weighing could be useful when applied to batches, where only the average weight would be of interest. By contrast, it could be difficult to apply these models with a focus on estimated weights for individuals. In general, the weight estimates obtained by imaging still lack greater accuracy and precision, especially since in many cases the RMSEP value was above the standard deviation of the actual values. In this sense, the current study used a pure breed and an independent validation with external data (from different batches/farms), simulating on a smaller scale what would be expected in a practical application of weighing through three-dimensional images. This section may be divided by subheadings. It should provide a concise and precise description of the experimental results, their interpretation, as well as the experimental conclusions that can be drawn.

## 4. Conclusions

The use of three-dimensional images has the potential to predict BW and HCW in Nellore bulls and steers. The biometric measurements obtained through a three-dimensional images system may help to monitor cattle growth and performance.

The use of independent validation helped to reduce the overfitting effect of the model, but future studies with a larger database and animals from different farms will still be necessary in order to offer greater robustness and precision in the predictions.

## Figures and Tables

**Table 1:** Descriptive statistics of body weights at the end of the feedlot period and hot carcass (in kg) of Nellore cattle in four different experiments (Exp.), as well as the sets formed for training predictive models.

	Training								Validation (Test)						
		n	Mean (kg)	SD ^1^ (kg)	CV ^2^ (%)	Min (kg)	Max (kg)		n	Age ^3^	Mean (kg)	SD ^1^ (kg)	CV ^2^ (%)	Min (kg)	Max (kg)
BW	Set 1	359	553.30	63.08	11.40	359.00	665.00	Exp. 4	91	24 ± 2	589.94	30.26	5.12	505.00	653.00
	Set 2	222	532.18	65.90	12.38	359.00	653.00	Exp. 3	228	22 ± 2	558.49	35.61	6.05	485.00	665.00
	Set 3	367	570.67	59.30	10.39	359.00	665.00	Exp. 2	83	22 ± 2	516.69	38.22	7.39	450.00	612.00
	Set 4	402	573.99	45.59	7.94	450.00	665.00	Exp. 1	48	22 ± 2	449.48	47.46	10.55	359.00	554.00
HCW	Set 1	359	315.34	37.32	11.83	205.00	394.00	Exp. 4	91	24 ± 2	334.22	16.79	5.02	277.60	376.80
	Set 2	222	301.70	36.42	12.07	205.00	376.80	Exp. 3	228	22 ± 2	336.17	23.17	6.92	259.20	394.40
	Set 3	367	325.74	34.02	10.44	204.80	394.40	Exp. 2	83	22 ± 2	290.08	22.19	7.65	248.80	343.60
	Set 4	402	326.21	28.49	8.73	248.80	394.40	Exp. 1	48	22 ± 2	260.12	28.10	10.80	205.00	317.00

BW = Body weight; HCW = Hot carcass weight; ^1^ Standard deviation; ^2^ Coefficient of variation; ^3^ Months.

**Table 2:** Evaluation parameters of body weight predictions in different experimental sets.

Training Dataset/Test	Models	Slope (±SE) ^1^	H0: b = 1	*r* ^2^	Mean Bias (kg)	CCC ^2^	RMSEP (kg) ^3^	RMSEP/Mean (%)	Decomposition of MSEP (%)		
									Mean Bias	Slope Bias	Random Error
Set 1/ Exp. 4	ANN	0.59 (±0.05)	0.01	0.58	3.22	0.73 (±0.04)	19.68	3.34	2.68	37.90	59.42
	PLS	0.69 (±0.06)	0.01	0.55	5.33	0.72 (±0.04)	21.60	3.66	6.09	18.11	75.80
	LASSO	0.70 (±0.06)	0.02	0.58	6.52	0.74 (±0.04)	20.95	3.55	9.71	18.55	71.74
	MLR	0.69 (±0.06)	0.01	0.58	6.62	0.74 (±0.04)	20.95	3.55	10.00	18.95	71.05
Set 2/ Exp. 3	ANN	0.57 (±0.03)	0.01	0.53	−12.19	0.66 (±0.03)	27.22	5.11	20.05	30.33	49.62
	PLS	0.59 (±0.03)	0.01	0.53	−13.80	0.65 (±0.03)	28.07	5.03	24.18	26.17	49.65
	LASSO	0.60 (±0.03)	0.01	0.53	−14.12	0.65 (±0.03)	28.31	5.07	24.90	24.70	50.40
	MLR	0.59 (±0.03)	0.01	0.53	−13.55	0.66 (±0.03)	27.95	5.00	23.53	25.86	50.61
Set 3/ Exp. 2	ANN	0.62 (±0.06)	0.01	0.53	6.04	0.70 (±0.05)	27.23	5.27	4.91	27.11	67.98
	PLS	0.56 (±0.09)	0.02	0.30	−0.65	0.54 (±0.07)	36.87	7.14	0.03	20.22	79.75
	LASSO	0.58 (±0.08)	0.01	0.39	5.81	0.61 (±0.06)	32.34	6.26	3.23	24.05	72.72
	MLR	0.56 (±0.07)	0.01	0.39	1.96	0.62 (±0.06)	31.36	6.07	0.40	27.65	71.95
Set 4/ Exp. 1	ANN	0.74 (±0.09)	0.08	0.59	12.40	0.74 (±0.06)	33.75	5.87	12.51	12.14	74.35
	PLS	0.78 (±0.11)	0.06	0.51	11.88	0.69 (±0.07)	39.34	8.75	9.12	6.35	84.53
	LASSO	0.76 (±0.11)	0.04	0.50	16.77	0.67 (±0.07)	40.63	9.04	17.04	7.33	75.63
	MLR	0.72 (±0.06)	0.04	0.51	13.77	0.67 (±0.07)	42.65	9.49	7.35	17.06	75.59

^1^ SE = standard error; ^2^ CCC = concordance correlation coefficient; ^3^ RMSEP = root mean square error prediction; SE = error standard; MSEP = mean square error of predict; ANN = artificial neural network; PLS = partial least squares; MLR = multiple linear regression.

**Table 3:** Evaluation parameters of hot carcass weight predictions in different experimental sets.

Training Dataset/Test	Models	Slope (±SE) ^1^	H0:b = 1	*r* ^2^	Mean Bias (kg)	CCC ^2^	RMSEP (kg) ^3^	RMSEP/ Mean (%)	Decomposition of MSEP (%)		
									Mean Bias	Slope Bias	Random Error
Set 1/ Exp. 4	ANN	0.56 (±0.08)	0.01	0.45	3.16	0.65 (±0.05)	13.06	3.90	5.87	30.78	63.35
	PLS	0.59 (±0.06)	0.01	0.44	6.89	0.60 (±0.06)	14.62	4.37	22.23	21.77	56.00
	LASSO	0.63 (±0.06)	0.01	0.49	4.43	0.67 (±0.05)	13.17	3.94	11.31	21.37	67.32
	MLR	0.08 (±0.03)	0.01	0.05	0.01	0.14 (±0.06)	8.75	2.62	0.00	86.95	13.05
Set 2/ Exp. 3	ANN	0.19 (±0.03)	0.01	0.17	−21.27	0.19 (±0.03)	30.00	8.92	50.25	38.55	11.20
	PLS	0.44 (±0.03)	0.01	0.42	−10.06	0.54 (±0.03)	20.33	6.05	24.49	39.51	36.00
	LASSO	0.43 (±0.03)	0.01	0.40	−9.11	0.53 (±0.03)	20.13	5.99	20.50	42.12	37.38
	MLR	0.43 (±0.03)	0.01	0.40	−9.51	0.53 (±0.03)	20.29	6.04	21.96	41.55	36.49
Set 3/ Exp. 2	ANN	0.52 (±0.06)	0.01	0.44	11.33	0.56 (±0.06)	20.06	6.91	31.87	26.99	41.14
	PLS	0.57 (±0.09)	0.02	0.32	10.49	0.51 (±0.07)	23.01	7.93	20.77	16.57	62.66
	LASSO	0.55 (±0.06)	0.01	0.46	12.89	0.55 (±0.06)	20.89	7.20	38.10	22.31	39.59
	MLR	0.42 (±0.09)	0.01	0.20	9.82	0.40 (±0.08)	24.61	8.48	16.25	26.83	56.92
Set 4/ Exp. 1	ANN	0.61 (±0.08)	0.05	0.52	12.67	0.64 (±0.07)	23.20	8.91	29.83	21.14	49.03
	PLS	0.78 (±0.12)	0.09	0.45	5.18	0.65 (±0.08)	25.35	9.75	4.19	5.55	90.26
	LASSO	0.75 (±0.11)	0.03	0.48	8.53	0.66 (±0.07)	24.16	9.29	12.48	8.13	79.39
	MLR	0.75 (±0.10)	0.02	0.52	8.49	0.69 (±0.07)	22.76	8.75	13.93	8.91	77.16

^1^ SE = standard error; ^2^ CCC = concordance correlation coefficient; ^3^ RMSEP = root mean square error prediction; SE = error standard; MSEP = mean square error of predict; ANN = artificial neural network; PLS = partial least squares; MLR = multiple linear regression.

**Table 4:** Root mean square error prediction (RMSEP) calculated using the predictive models: ANN, PLS, LASSO, and RLM for body weight and hot carcass weight.

		Root Mean Square Error Prediction (RMSEP)							
		Body Weight (kg)				Hot Carcass Weight (kg)			
		Set 1	Set 2	Set 3	Set 4	Set 1	Set 2	Set 3	Set 4
Training set 10-kfold cross validation	ANN	20.16	20.91	18.69	20.27	15.71	13.42	15.25	14.82
	PLS	21.28	20.72	20.53	18.23	16.77	13.77	15.98	14.70
	LASSO	21.54	20.33	20.43	18.06	16.67	13.94	15.67	14.82
	RLM	23.62	21.83	22.21	21.00	7.33	13.95	15.04	15.08
Independent validation	ANN	19.68	27.22	27.23	33.75	13.06	30.00	20.06	23.20
	PLS	21.60	28.07	36.87	39.34	14.62	20.33	23.01	25.35
	LASSO	20.95	28.31	32.34	40.63	13.17	20.13	20.89	24.16
	RLM	20.95	27.95	31.36	40.63	8.75	20.29	24.61	22.76

SE = error standard; MSEP = mean square error of predict; ANN = artificial neural network; PLS = partial least squares; MLR = multiple linear regression.

## Data Availability

The data are available upon request from the corresponding author.

## Supplementary Materials

The following supporting information can be downloaded at: https://www.mdpi.com/article/10.3390/ani13101679/s1, Table S1. Models developed for artificial neural networks, partial least squares, and least absolute shrinkage and selection operator for body weight prediction; Table S2. Models developed for artificial neural networks, partial least squares, and least absolute shrinkage and selection operator to predict hot carcass weight.
