# Supplementary material for: Use of Biometric Images to Predict Body Weight and Hot Carcass Weight of Nellore Cattle
Source: Animals (Basel). 2023 May 18;13(10):1679. doi: 10.3390/ani13101679 (PMC10215216; doi:10.3390/ani13101679)
Supplement: Supplementary file 1 [file animals-13-01679-s001.zip › animals-2327520-supplementary.pdf]

animals

Supplementary Materials

Use of Biometric Images to Predict Body Weight and Hot Carcass Weight of Nellore Cattle

Alexandre Cominotte [1,2], Arthur Fernandes [1], João Dórea [1], Guilherme Rosa [1,3], Rodrigo Torres [4], Guilherme Pereira [4], Welder Baldassini [2,4] and Otávio Machado Neto [2,4,*]

[1] Department of Animal Science, University of Wisconsin, Madison, WI 53706, USA
[2] School of Agricultural and Veterinarian Sciences, São Paulo State University, Jaboticabal 14884-900, SP, Brazil
[3] Department of Biostatistics and Medical Informatics, University of Wisconsin, Madison, WI 53706, USA
[4] School of Veterinary and Animal Science, São Paulo State University, Botucatu 18618-681, SP, Brazil
* Correspondence: otavio.machado@unesp.br

Table S1. Models developed for Artificial Neural Networks, Partial Least Squares and Least absolute shrinkage and selection operator for body weight prediction.

Item	**Experimental sets** [1]			
	Set 1	Set 2	Set 3	Set 4
**ANN**				
1ªNeural Layers Unit	28	28	28	28
2° Type of Neural Layers	RectifierWithDropout	RectifierWithDropout	RectifierWithDropout	RectifierWithDropout
	100	100	100	100
Epoch	20	20	20	20
L1 [2]	0.000024	0.000024	0.000024	0.000024
L2 [3]	0.000019	0.000019	0.000019	0.000019
Input dropout ratio	0	0	0	0
hidden dropout reason	0	0	0	0
**PLS** [4]				
Number of Factors	8	5	8	13
**LASSO**				
Lambda	0.70	0.32	0.30	0.13

[1] Experimental sets: Set 1 = Experimental set composed of experiment 1, 2 and 3 for training and experiment 4 for validation of the predictive model; Set 2= Experimental set composed of experiment 1, 2 and 4 for training and experiment 3 for validation of the predictive model; Set 3 = Experimental set composed of experiment 1, 3 and 4 for training and experiment 2 for validation of the predictive model; Set 4 = Experimental set composed of experiment 2, 3 and 4 for training and experiment 1 for validation of the predictive model.
[2] L1 = Lasso Regularization.
[3] L2 = Ridge regularization.
[4] NIPALS method: Nonlinear interactive partial range algorithm used to calculate PLS regression components.

Table S2. Models developed for Artificial Neural Networks, Partial Least Squares and Least absolute shrinkage and selection operator to predict hot carcass weight

Item	Experiment set [1]			
	Set 1	Set 2	Set 3	Set 4
**ANN**				
1ªNeural Layers Unit	32	32	32	32
	RectifierWithDropout	RectifierWithDropout	RectifierWithDropout	RectifierWithDropout
2° Type of Neural Layers				
	100	100	100	100
Epoch	20	20	20	20
L1 [2]	0.000024	0.000024	0.000024	0.000024
L2 [3]	0.000019	0.000019	0.000019	0.000019
Input dropout ratio	0	0	0	0
Hidden dropout reason	0	0	0	0
**PLS** [4]				
Number of Factors	1	5	4	13
**LASSO**				
Lambda	0.57	1.80	0.16	0.18

[1]Experimental sets: Set 1 = Experimental set composed of experiment 1, 2 and 3 for training and experiment 4 for validation of the predictive model; Set 2 = Experimental set composed of experiment 1, 2 and 4 for training and experiment 3 for validation of the predictive model; Set 3 = Experimental set composed of experiment 1, 3 and 4 for training and experiment 2 for validation of the predictive model; Set 4 = Experimental set composed of experiment 2, 3 and 4 for training and experiment 1 for validation of the predictive model.
[2]L1 = Lasso Regularization.
[3]L2 = Ridge regularization.
[4]NIPALS method: Nonlinear interactive partial range algorithm used to calculate PLS regression components.
